# PATTERNS OF MULTIMORBIDITY IN AGING, RADIATION-EXPOSED NON-HUMAN PRIMATES

**DOI:** 10.1093/geroni/igac059.2935

**Published:** 2022-12-20

**Authors:** Ellen Quillen, Maggie Stainback, Jamie Justice, John Olson, George Schaaf, J Mark Cline

**Affiliations:** Wake Forest School of Medicine, Winston Salem, North Carolina, United States; Wake Forest School of Medicine, Winston Salem, North Carolina, United States; Wake Forest School of Medicine, Winston-Salem, North Carolina, United States; Wake Forest School of Medicine, Winston Salem, North Carolina, United States; Wake Forest School of Medicine, Winston Salem, North Carolina, United States; Wake Forest School of Medicine, Winston Salem, North Carolina, United States

## Abstract

Resilience to stressors is a major component of biological aging and may mediate the onset of multimorbidity in older adults. The Wake Forest Non-Human Primate Radiation Survivor Cohort (RSC) provides a novel opportunity to study aging and resilience in 250 rhesus macaques (Macaca mulatta) with single-dose radiation exposures 0–15 years prior and 50 controls with semi-annual clinical, imaging, and biomarker measurements taken over their lifespan. Multimorbidity is extremely common among irradiated animals. Only 38% of animals have none of 20 common chronic diseases, falling to 16% of animals over age 8 and 11% over age 10 (middle-aged animals). 70% of animals have 5 or more diagnoses in this oldest cohort. The presence of any one disease increases the likelihood of having a second, co-morbid condition. Nevertheless, some animals continue disease-free until late in life, highlighting substantial variability in resilience. To identify patterns of multimorbidity, survival curves for each diagnosis were generated for age and time since radiation and k-median clustered resulting in four groupings of aging-associated morbidities. Bone, brain, and gastrointestinal disorders arise 3.5 years after radiation on average, followed by skin, heart, and cataracts. At 4.65 years, animals are at increased risk of being underweight and overweight and developing diabetes, hypertension, and hepatic dysfunction. Tumor, lung, and kidney disorders arise approximately 6 years after exposure. In all cases, these age-related disorders occur significantly earlier in irradiated animals than controls. These findings highlight the clustering of multimorbidities in aging, radiation-challenged primates and the potential of the RSC in studying resilience.

